# Synthesis and Antiviral Bioactivity of Chiral Thioureas Containing Leucine and Phosphonate Moieties 

**DOI:** 10.3390/molecules15085112

**Published:** 2010-07-29

**Authors:** Jingzi Liu, Song Yang, Xiangyang Li, Huitao Fan, Pinaki Bhadury, Weiming Xu, Jian Wu, Zhencao Wang

**Affiliations:** 1 State Key Laboratory Breeding Base of Green Pesticide and Agricultural Bioengineering, Key Laboratory of Green Pesticide and Agricultural Bioengineering, Ministry of Education, Guizhou University, Guiyang 550025, China; E-Mails: liujingz@126.com (J.L.); michael.lee1983@yahoo.com.cn (X.L.); fanhuitao0818@163.com (H.F.); bhadury@gzu.edu.cn (P.B.); xuweiming2009@163.com (W.X); jianwu2691@yahoo.com.cn (J.W); wzc.4884@163.com (Z.W); 2 Laboratory of Medicinal Chemistry, Zunyi Medical College, Zunyi, 563003, China

**Keywords:** chiral thiourea, leucine, phosphonate, antiviral activity, synthesis

## Abstract

A series of novel chiral thioureas **3a-n **bearing leucine and phosphonate moieties were synthesized in excellent yields. The structures of the compounds were completely characterized by elemental analysis, IR,^ 1^H-, ^13^C-, ^31^P- and ^19^F-NMR spectral data. A half-leaf method was used to determine the *in vivo* protective and curative efficacies of the title products against tobacco mosaic virus (TMV). The compounds **3l** and **3n** displayed good *in vivo* protection and curative effects against TMV with inhibitory rates of 60.1, 62.8% (protection) and 56.7, 53.6% (curative) at 0.5 mg/mL, respectively. To the best of our knowledge, this is the first report on the antiviral activity of chiral thioureas containing leucine and phosphonate moieties.

## 1. Introduction

**Scheme 1:**
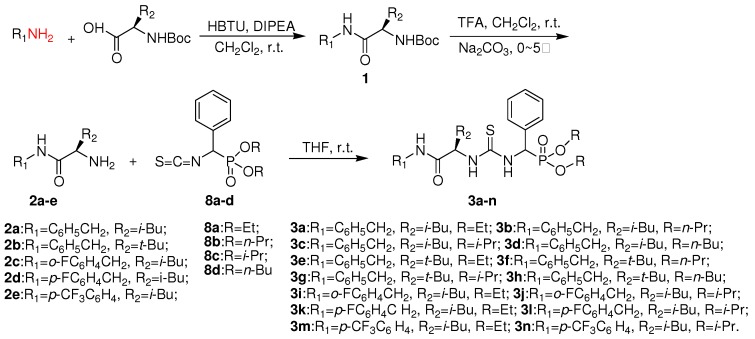
Synthetic route to chiral thiourea derivatives **3a-n**.

Chiral thioureas and their derivatives constitute an important class of compounds which exhibit a wide range of antibacterial, fungicidal, herbicidal, antiviral and plant growth regulatory activities. Some of them can serve not only as chiral organocatalysts in the synthesis of asymmetric compounds [1], but also as potential anticancer and anti-HIV drugs [2,3,4]. Amongst the noteworthy contributions reported in this field, Venkatachalam and his group [4] obtained chiral naphthyl thioureas (CNT) as potent non-nucleoside inhibitors (NNI) of the reverse transcriptase (RT) enzyme of HIV-1. Interestingly, the *R-*enantiomers of these derivatives could inhibit the recombinant RT *in vitro* with lower IC_50_ values compared to their corresponding *S-*enantiomers. Unfortunately, chiral thiourea derivatives have scarcely been evaluated for their plant antiviral activities for agricultural applications. It is well known that certain bioisosteres of natural amino acids and *a*-aminophosphonic acid analogues are associated with significant bioactivities and are often employed as enzyme inhibitors, antimicrobial, antitumor and antiviral agents [5,6,7,8,9]. Further, suitably substituted phosphonic analogues of L-leucine can play prominent role in leucine aminopeptidase inhibition and in the early stages of HIV infection [10,11]. In this context, we have previously discovered some novel *α*-aminophosphonate derivatives with appreciable antiviral activity against the tobacco mosaic virus (TMV) [12,13,14]. Based on these findings, we turned our attention to the incorporation of the thiourea and *α*-aminophosphonate pharmacophores into a single structure through a chiral L*-*leucine linker. The idea was to generate new potentially antiviral chiral thiourea derivatives **3a-n **with high activity, low toxicity and minimal residues [15]. The synthetic route is shown in Scheme 1. The structures of the target compounds were firmly established by IR, ^1^H-, ^13^C-, ^31^P- and ^19^F-NMR spectra and elemental analysis. Preliminary bioassay tests showed that some of these compounds possessed good *in vivo* anti-TMV activities. 

## 2. Results and Discussion

### 2.1. Chemistry

**Scheme 2:**
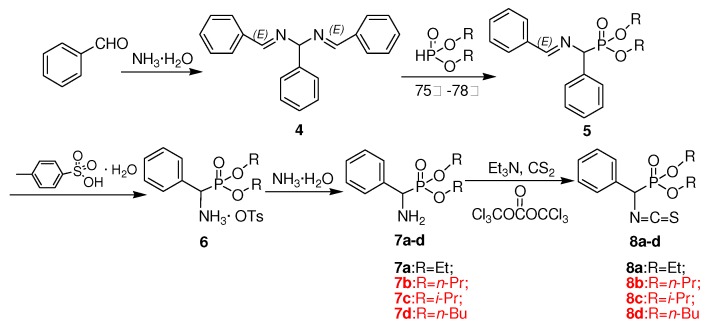
Synthetic route to intermediates **8a-d**.

The synthetic route designed for the chiral thiourea analogues **3a-n** containing L-leucine and an *α*-aminophosphonate moiety is summarized in Scheme 1. Boc-protected intermediate amide **1** was first generated from the reaction of L-*N*-Boc-leucine and a substituted arylamine in the presence of *O*-benzotriazol-1-yl-*N,N,N’,N’*-tetramethyluronium hexafluorophosphate (HBTU) at room temperature. The *α*-amino carboxamide derivatives **2a-e** were obtained through Boc deprotection using trifluoroacetic acid, as shown in Scheme 1. The desired chiral thiourea analogues **3a-n** were then prepared by the addition of *O,O′*-dialkylisothiocyanato(phenyl)methylphosphonates **8a-d** to **2a-e** in THF at room temperature. The intermediates **8** were in turn obtained from benzaldehyde in five steps by following a literature procedure [13], as shown in Scheme 2. 

The existing method for the conversion of product **7** into **8** by the use of phosphorus oxychloride (POCl_3_) was however not satisfactory. This step of the reaction was further optimized by employing different reagents, such as bis(trichloromethyl) carbonate (BTC), thiophosgene (CSCl_2_) and 1,1-thiocarbonyldiimidazole (TCDI). The results of these experiments were compared with those obtained with the phosphorus oxychloride method (Table 1). As could be observed from the data, the best result was obtained with BTC, which afforded 67-79% isolated yield of **8a-8d** against the reported yield [13] of 54-62%. Therefore, BTC was selected as the ideal reagent for this key conversion.

**Table 1:** Comparison of the yield for intermediates **8a-d** using different reagents.

Compd.	R	Yield (%)			
		CSCl_2_	TCDI	BTC	POCl^a^
**8a**	Et	24	trace	79	60
**8b**	*n*-Pr			71	62
**8c**	*i*-Pr			67	54
**8d**	*n*-Bu			75	58

^a ^Reported yields in the reference [13].

With regard to the best solvent that could be used for the preparation of title compounds **3** from **2** and **8 **(Scheme 1), the model compound **3e **was synthesized in different solvents, e.g. tetrahydrofuran (THF), acetonitrile (CH_3_CN), acetone (CH_3_COCH_3_), dichloromethane (CH_2_Cl_2_) and toluene and the results are provided in Table 2. Under optimized conditions, a maximum yield of 98% could be obtained when the reaction mixture was stirred at room temperature in the solvent THF for 0.5 h. 

**Table 2:** Role of different solvents for the synthesis of **3e**.

No.	Solvent	Vol. (mL)	Reaction time (h)	Temperature (ºC)	Yield (%)
**1**	THF	5	0.5	room	98
**2**	CH_3_CN	5	0.5	room	76
**3**	CH_3_COCH_3_	5	0.5	room	89
**4**	CH_2_Cl_2_	5	0.5	room	73
**5**	toluene	5	0.5	room	55.7

All the products were unequivocally characterized by IR and NMR spectral data and elemental analyses. The characteristic IR absorption bands at 3269-3467 cm^-1^, 1645-1678 cm^-1^, 1546-1564 cm^-1^, 1212-1227 cm^-1^, and 1008-1028 cm^-1 ^confirmed the presence of NH, C=O, C=S, P=O and P-O-C functional groups, respectively. In the ^1^H-NMR spectra of title compounds **3a-n**, all aromatic protons displyed the expected multiplet near 6.78-7.79 ppm. While the amidic NH protons appeared downﬁeld in the 7.74-10.12 ppm range as broad singlets, the NH protons of thioureido groups revealed weak peaks due to the existence of hydrogen bonds. The typical phosphorus resonance at 19.7-22.8 ppm in the ^31^P-NMR spectra of all target compounds confirmed the presence of a phosphorus center coupled to an adjacent CH. In the ^19^F-NMR spectra, the fluorine resonance due to Ar-F and CF_3_ appeared at ‑115.1 to -118.8 ppm and -63.2 ppm, respectively. The typical carbon resonances at 183.1-184.6 and 170.6-174.4 ppm in the ^13^C-NMR spectra of the title compounds were indicative of the existence of C=S and C=O double bonds, respectively. All the nonequivalent carbon atoms were identified in the ^13^C-NMR and the total number of protons calculated from the ^1^H-NMR integration curves was in complete agreement with the assigned structures.

### 2.2. Antiviral activity

The results of the *in vivo* bioassay against TMV are given in Table 3. Ningnanmycin was used as the reference antiviral agent. Among the studied compounds, thiourea derivatives **3l** (R_1_= *p*-FC_6_H_4_CH_2_, R_2_= *i*-Bu, R= *i*-Pr) and **3n** (R_1_= *p*-CF_3_C_6_H_4_, R_2_= *i*-Bu, R= *i*-Pr) displayed remarkable protection activities (60.1 and 62.8%, respectively) and curative rates (56.7 and 53.6%, respectively) against TMV, which were comparable to the protection activity (53.1%) and curative rate (55.3%) shown by the commercial reference ningnanmycin. The results indicated that the antiviral activity depended to a certain extent on the type of stereochemical configuration and the nature of the substituents present in the compound. In particular, the title compounds derived from *iso*-leucine (R_2_= *i*-Bu) showed better anti-TMV activity compared to those derived from *tert*-leucine (R_2_= *tert*-Bu). Thus, the following trend in curative trend was noted: **3a** (42.3%) > **3e** (28.4%), **3b** (35.4%) > **3f** (33.5%), **3c** (46.2%) > **3g** (40.9%) and **3d** (31.2%) > **3h** (24.1%). Furthermore, *iso*-leucine-derived thioureas incorporating *O,O’*-diisopropylphosphonate (R= *i*-Pr) were found to be superior in terms of anti-TMV activity compared to their phosphonate analogues which were obtained from diethyl, di-*n*-propyl or di-*n*-butyl phosphites (R= Et, *n*-Pr, *n*-Bu). Evidently, a phosphonate moiety derived from branched alkyl chains should be the preferred over straight chain alkylated ones in the design of the title thioureas. In addition, the compounds bearing an electron withdrawing group (R_1_= *p*-F,*p*-CF_3_)at the 4-position of the aromatic ring showed better anti-TMV activities, with inhibition rates ranging from 48.1 to 56.7%. At for the absolute configuration, it was observed that the enantiomer D-**3l** and racemate (±)-**3l** did not reveal any noticeable anti-TMV activity (curative rates 28.4% and 34.9%, respectively). This was in sharp contrast to L*-***3l** (derived fromL-leucine) which showed significant activity against TMV. In order to establish a definite structure-activity relationship, some structural modification needs to be conducted by making subtle changes in the nature of substituents of title thioureas having L*-*configuration.

**Table 3:** The protection effect and curative effect of the compounds **3a-n **against TMV *in vivo.*

Compd.	R_1_	R_2_	R	Concentration (mg/mL)	Protection Effect (%)	Curative Effect (%)
**3a**	C_6_H_5_CH_2_	*i*-Bu	Et	0.5	19.6*	42.3*
**3b**	C_6_H_5_CH_2_	*i*-Bu	*n*-Pr	0.5	27.5*	35.4*
**3c**	C_6_H_5_CH_2_	*i*-Bu	*i*-Pr	0.5	52.8**	46.2*
**3d**	C_6_H_5_CH_2_	*i*-Bu	*n*-Bu	0.5	12.3*	31.2*
**3e**	C_6_H_5_CH_2_	*tert*-Bu	Et	0.5	34.0*	28.4*
**3f**	C_6_H_5_CH_2_	*tert*-Bu	*n*-Pr	0.5	45.1*	33.5*
**3g**	C_6_H_5_CH_2_	*tert*-Bu	*i*-Pr	0.5	23.4*	40.9*
**3h**	C_6_H_5_CH_2_	*tert*-Bu	*n*-Bu	0.5	9.8*	24.1*
**3i**	*o*-FC_6_H_4_CH_2_	*i*-Bu	Et	0.5	43.4*	45.3*
**3j**	*o*-FC_6_H_4_CH_2_	*i*-Bu	*i*-Pr	0.5	48.3**	51.3**
**3k**	*p*-FC_6_H_4_CH_2_	*i*-Bu	Et	0.5	44.9*	48.1**
**3l**	*p*-FC_6_H_4_CH_2_	*i*-Bu	*i*-Pr	0.5	60.1**	56.7**
***D*-3l**	*p*-FC_6_H_4_CH_2_	*i*-Bu	*i*-Pr	0.5	–	28.4*
**±3l**	*p*-FC_6_H_4_CH_2_	*i*-Bu	*i*-Pr	0.5	–	34.9*
**3m**	*p*-CF_3_C_6_H_4_	*i*-Bu	Et	0.5	51.2*	49.8**
**3n**	*p*-CF_3_C_6_H_4_	*i*-Bu	*i*-Pr	0.5	62.8**	53.6*
**Ningnanmycin**	–	–	–	0.5	53.1**	55.3*

n = 3 for all groups; * P < 0.05, ** P < 0.01.

## 3. Experimental

### 3.1. General

The melting points of the products were determined on a XT-4 binocular microscope (Beijing Tech Instrument Co., China) and were not corrected. The IR spectra were recorded on a Bruker VECTOR22 spectrometer in KBr disks. The^1^H, ^13^C,^19 ^F and ^31^P NMR spectra (solvent DMSO-*d*_6_ or CDCl_3_) were recorded at room temperature on a JEOL-ECX 500 NMR spectrometer operating at 500, 125, 470 and 200 MHz, respectively, using TMS as an internal standard. Data are reported as follows: chemical shifts in ppm (*δ*), multiplicity (s = singlet, d = doublet, t = triplet, q = quartet, br = broad and m = multiplet, coupling constant (Hz) and integration. Elemental analyses were performed on an Elementar Vario-III CHN analyzer. The reagents were all of analytical grade or chemically pure. Analytical TLC was performed on silica gel GF254 plates.

### 3.2. Preparation of intermediates 2a-e

L-*N*-Boc-leucine (2.31 g, 0.01 mol) and *O*-benzotriazol-1-yl-*N,N,N’,N’*-tetramethyluronium hexa- fluorophosphate (HBTU, 3.80 g, 0.01 mol) were loaded into an oven-dried round bottomed flask equipped with a magnetic stir bar, rubber septum, and argon inlet. Anhydrous dichloromethane (50 mL) was then added. After 3 minutes, anhydrous DIPEA (2.58 g, 0.02 mol) and arylamine (0.011 mol) were sequentially added and the reaction mixture was stirred at room temperature for 2-4 h. During the process, the state of the solution was seen to change from slightly heterogeneous to homogeneous confirming the consumption of HBTU. The reaction mixture was poured into a separatory funnel containing 1N HCl (50 mL), and was then partitioned between dichloromethane and aqueous hydrochloric acid solution. The organic layer was washed with 1N HCl (3 × 30 mL), dried over anhydrous sodium sulfate, filtered, and concentrated *in vacuo*. The resulting light yellow oil was transferred to a 100-mL flask and redissolved in dichloromethane (30 mL). Trifluoroacetic acid (7 mL) was then added in one portion. After 2-4 h, the mixture was slowly partitioned with dichloromethane and chilled, saturated aqueous sodium carbonate solution (50 mL) was added. The aqueous layer was extracted with dichloromethane (3 × 30 mL), and the combined organic extracts were dried over anhydrous sodium sulfate, filtered, and concentrated *in vacuo*. The crude product was purified by thin layer chromatography (TLC) on a silica gel (developing solvent: 3-4% MeOH/CH_2_Cl_2_, *V/V*) to give the intermediates **2a-e**. 

*L-2-Amino-N-benzyl-4-methylpentanamide *(**2a**). White crystals, yield 77%, m.p. 60-62 ºC; ^1^H-NMR (CDC1_3_): *δ* 0.95 (d, 6H, *J* = 3.2 Hz, 2CH_3_), 1.73-1.75 (m, 2H, CH_2_), 1.96 (br s, 2H, NH_2_), 2.80-2.82 (m, 1H, CH), 3.45-3.48 (m, H, CH), 4.43 (d, *J* = 6.3 Hz, 2H, NCH_2_), 7.26-7.35 (m, 5H, ArH), 7.72 (br s, 1H, NH); ^13^C-NMR (CDC1_3_): *δ* 175.6, 138.6, 128.7, 127.8, 127.4, 53.6, 44.1, 43.2, 24.9, 21.4; IR (KBr, cm^-1^): *v* 3304, 2958, 2868, 1647; Anal. Calcd. for C_13_H_20_N_2_O: C 70.87, H 9.15, N 12.72; Found: C 70.62, H 9.34, N 12.86.

*L-2-Aamino-N-benzyl-3,3-dimethylbutanamide* (**2b**). White crystals, yield 83%, m.p. 53-54 ºC, ^1^H-NMR (CDC1_3, _500 MHz): *δ* 0.99 (s, 9H, 3CH_3_), 1.56 (br s, 2H, NH_2_), 3.11 (s, 1H, CH), 4.42 (d, *J* = 3.45 Hz, 2H, NCH_2_), 7.25-7.31 (m, 5H, ArH), 7.78 (br s, 1H, NH); ^13^C-NMR (CDC1_3__,_ 125 MHz): *δ* 173.6, 138.6, 128.7, 128.0, 127.5, 64.5, 43.2, 34.3, 26.9; IR (KBr, cm^-1^): *v* 3306, 2946, 1650; Anal. Calcd. for C_13_H_20_N_2_O: C 70.87, H 9.15, N 12.72; Found: C 70.71, H 9.23, N 12.36. 

*L-N-(2-Fluorobenzyl)-2-amino-4-methylpentanamide* (**2c**). Colorless viscous liquid, yield 68%; ^1^H-NMR (CDC1_3_): *δ* 0.99 (d, 6H, *J* = 4.6 Hz, 2CH_3_), 1.23 (t, 2H, *J* = 7.2 Hz, CH_2_), 1.65 (br s, 2H, NH_2_), 1.76-1.84 (m, 1H, CH), 3.52-3.55 (m, H, CH), 4.23 (d, *J* = 5.3 Hz, 2H, NCH_2_), 7.05-7.33(m, 4H, ArH), 8.43 (br s, 1H, NH); ^13^C-NMR (CDC1_3_): *δ* 174.2, 159.7, 131.4, 128.8, 115.3, 124.1, 53.2, 43.5, 34.6, 24.6, 21.3; ^19^F-NMR (CDC1_3_): *δ* -115.5; IR (KBr, cm^-1^) *v*: 3308, 2960, 2873, 1649; Anal. Calcd. for C_13_H_19_FN_2_O: C 65.52, H 8.04, N 11.76; Found: C 65.36, H 8.32, N 11.65. 

*L-N-(4-Fluorobenzyl)-2-amino-4-methylpentanamide *(**2d**). Colorless liquid, yield 73%, *n*^25^_*D*_ 1.5055; ^1^H-NMR (CDC1_3_): *δ* 0.90 (d, 6H, *J* = 3.4 Hz, 2CH_3_), 1.63-1.72 (m, 2H, CH_2_), 2.25 (br s, 2H, NH_2_), 2.76-2.79 (m, 1H, CH), 3.35-3.38 (m, 2H, CH), 4.34 (d, *J* = 5.15 Hz, 2H, NCH_2_), 6.96-7.22 (m, 4H, ArH), 7.98 (br s, 1H, NH); ^13^C-NMR (CDC1_3_): *δ* 174.7, 161.5, 137.4, 128.6, 115.2, 52.9, 44.5, 42.8, 24.7, 21.4; ^19^F-NMR (CDC1_3_): *δ* -115.3; IR (KBr, cm^-1^): *v* 3309, 2954, 2870, 1645, 1504, 1223; Anal. Calcd. for C_13_H_19_FN_2_O: C 65.52, H 8.04, N 11.76; Found: C 65.33, H 8.26, N 11.51.

*L-2-Amino-4-methyl-N-(4-(trifluoromethyl)phenyl)pentanamide *(**2e**). Colorless liquid, yield 56%, *n*^25^_*D*_ 1.4750; ^1^H-NMR (CDC1_3_): *δ* 0.95 (d, 6H, *J* = 6.3 Hz, 2CH_3_), 1.43-1.47 (m, 1H, CH), 1.75-1.79 (m, 1H, CH_2_), 2.51 (br s, 2H, NH_2_), 3.53 (d, 1H,*J* = 4.0 Hz, , NCH), 7.54-7.78(d, 4H, *J* = 8.6 Hz, ArH), 9.99 (br s, 1H, NH); ^13^C-NMR (CDC1_3_): *δ* 174.7, 141.3, 125.9, 125.6, 123.3, 119.1, 54.0, 43.7, 24.8, 21.4; ^19^F-NMR (CDC1_3_): *δ* -62.0; IR (KBr, cm^-1^): *v* 3462, 3269, 2953, 1628, 1506, 1323, 1115, 1067; Anal. Calcd. for C_13_H_17_F_3_N_2_O: C 56.93, H 6.25, N 10.21; Found: C 57.18, H 6.47, N 10.39. 

### 3.3. Preparation of O,O′-dialkylisothiocyanato(phenyl)methylphosphonate intermediates **8a-d**

To a solution of *α*-aminophosphonate (6 mmol) in ether (15 mL), triethylamine (18 mmol) was added with constant stirring at room temperature and cooled to 0 ºC. Then, carbon disulfide (6 mmol) was added dropwise and the mixture stirred for 1 h at 0 ºC, the temperature was raised to 23 ºC, and stirring was continued for an additional 2 h. BTC (2 mmol) dissolved in ether (10 mL) was then added dropwise into the reaction mixture and stirred for 3 h at 23 ºC. The solid was filtered off, and the liquid was extracted with ether, treated with saturated sodium bicarbonate, and dried on anhydrous sodium sulfate, filtered. Removal of the solvent followed by chromatography of the crude product on silica using a mixture of petroleum ether and ethyl acetate as the developing solvent gave the intermediates **8** in 67-79% yields; data for **8a-d** can be found in reference [13].

### 3.4. Preparation of title chiral thioureas **3a-n**

A solution of *O,O’*-dialkylisothiocyanato(phenyl)methylphosphonate **8 **(1 mmol) in tetrahydrofuran (5 mL) was stirred, followed by dropwise addition of chiral amine **2a-e** (1.1 mmol). The stirring was continued for 0.5 h at 23 ºC, the solvent was evaporated and the crude product was purified by preparative TLC using a mixture of ethyl acetate and *n*-hexane (*V*:*V *= 1:1) as developing solvent to give title compound **3a-n**. 

*Diethyl (3-(L-1-benzylamino-4-methyl-1-oxopentan-2-yl)thioureido)(phenyl)methylphosphonate* (**3a**). White solid, yield 94%; m.p. 43-45 ºC; [*α*]^20^*_D_*-17.9º (*c *0.1, acetone); ^1^H-NMR (CDC1_3__,_ 500 MHz): *δ* 0.79 (d, 6H, *J* = 6.3 Hz, 2CH_3_), 1.06 (t, 6H, *J* = 5.75 Hz, 2CH_3_), 1.24-1.46(m, 2H, CH_2_), 1.65-1.72 (m, 1H, CH), 3.68-4.07 (m, 2H, NCH_2_), 4.12-4.49 (m, 4H, 2OCH_2_) , 5.07 (d, 1H, *J *= 6.7 Hz, CH), 6.28 (s, 1H, PCH), 6.45 (s, 1H, NH), 6.95-7.48 (m, 10H, ArH), 7.91 (s, 1H, NH), 8.82 (br s, 1H, NH); ^13^C-NMR (CDCl_3__,_ 125 MHz): *δ* 183.1, 172.5, 139.2, 136.1, 128.5, 127.6, 126.9, 64.3, 58.1, 54.3, 44.5, 41.4, 26.4, 23.7, 16.3; ^31^P- NMR (CDCl_3__,_ 200 MHz):*δ* 21.8; IR (KBr, cm^-1^): *v* 3284, 3065, 2938, 1659, 1560, 1216, 1012; Anal. Calcd for C_25_H_36_N_3_O_4_PS: C 59.39, H 7.18, N 8.31; Found C 59.57, H 7.33, N 8.15.

*Dipropyl (3-(L-1-benzylamino-4-methyl-1-oxopentan-2-yl)thioureido)(phenyl)methylphosphonate* (**3b**). Colorless viscous liquid, yield 89%; [*α*]^20^*_D_*-15.8º (*c *0.1, acetone); ^1^H-NMR (CDC1_3_): *δ* 0.81-1.04 (m, 12H, 4CH_3_), 1.27-1.45 (m, 6H, 3CH_2_), 1.67-1.70 (m, 1H, CH), 3.71-4.09 (m, 2H, NCH_2_), 4.12-4.53 (m, 4H, 2OCH_2_), 5.10 (d, 1H, *J *= 6.4 Hz, CH), 6.31 (s, 1H, PCH), 6.48 (s, 1H, NH), 6.94-7.49(m, 10H, ArH), 7.95 (s, 1H, NH), 9.07 (br s, 1H, NH); ^13^C-NMR (CDC1_3_): *δ* 183.5, 172.3, 138.4, 135.6, 128.8, 127.1, 126.7, 68.2, 62.5, 54.4, 43.8, 41.3, 26.5, 24.2, 22.7, 10.1; ^31^P-NMR (CDCl_3_): *δ* 21.5; IR (KBr, cm^-1^): 3276, 3081, 2927, 1663, 1564, 1219, 1009 cm^-1^; Anal. Calcd for C_27_H_40_N_3_O_4_PS: C 60.77, H 7.55, N 7.87; Found: C 60.52, H 7.81, N 7.69.

*Diisopropyl (3-(L-1-benzylamino-4-methyl-1-oxopentan-2-yl)thioureido)(phenyl)methylphosphonate* (**3c**). White solid, yield 95%; m.p. 51-53 ºC; [*α*]^20^*_D_*-14.2º (*c *0.1, acetone); ^1^H-NMR (CDC1_3_): *δ* 0.82 (d, 18H, *J *= 6.3 Hz, 6CH_3_), 1.42-1.59 (m, 2H, CH_2_), 1.76-1.89 (m, H, CH), 4.26-4.48 (m, 2H, NCH_2_), 4.69-4.78 (m, 2H, 2OCH), 5.09 (d, 1H, *J *= 5.75 Hz, CH), 6.27 (s, 1H, PCH), 7.06-7.46 (m, 10H, ArH), 7.81 (s, 1H, NH), 8.73 (br s, 1H, NH); ^13^C-NMR (CDC1_3_): *δ* 183.9, 172.0, 138.2, 135.5, 128.7, 128.5, 127.6, 127.2, 73.2, 56.4, 55.9, 43.5, 40.2, 38.7, 24.3, 23.1; ^31^P-NMR (CDCl_3_): *δ* 22.8; IR (KBr, cm^-1^): *v* 3285, 3063, 2929, 1674, 1558, 1215, 1014; Anal. Calcd for C_27_H_40_N_3_O_4_PS: C 60.77, H 7.55, N 7.87; Found: C 60.63, H 7.24, N 7.66.

*Dibutyl (3-(L-1-benzylamino-4-methyl-1-oxopentan-2-yl)thioureido)(phenyl)methylphosphonate* (**3d**). Colorless viscous liquid, yield 86%; [*α*]^20^*_D_*-16.4º (*c* 0.1, acetone); ^1^H-NMR (CDC1_3_): *δ* 0.80-0.95 (m, 12H, 4CH_3_), 1.20-1.43 (m, 10H, 5CH_2_), 1.55-1.72 (m, 1H, CH), 3.66-4.05 (m, 2H, NCH_2_), 4.11-4.55 (m, 4H, 2OCH_2_), 5.04 (d, 1H, *J *= 6.3 Hz, CH), 6.27 (s, 1H, PCH), 6.43(s, 1H, NH), 6.92-7.44 (m, 10H, ArH), 7.83 (s, 1H, NH), 8.70 (br s, 1H, NH); ^13^C-NMR (CDC1_3_): *δ* 184.5, 170.6, 138.2, 135.6, 128.9, 128.6, 127.8, 67.7, 65.8, 55.5, 43.4, 34.8, 32.2, 26.9, 18.8, 13.7; ^31^P-NMR (CDCl_3_): *δ* 22.1; IR (KBr, cm^-1^): *v* 3269, 3082, 2953, 1670, 1550, 1220, 1024; Anal. Calcd for C_29_H_44_N_3_O_4_PS: C, 62.01; H, 7.90; N, 7.48; Found: C 62.28, H 7.67, N 7.72.

*Diethyl (3-(L-1-benzylamino-3,3-dimethyl-1-oxobutan-2-yl)thioureido)(phenyl)methylphosphonate* (**3e**). White crystals, yield 98%; m.p. 157-159 ºC; [*α*]^20^*_D_*+15.5º (*c *0.1, acetone); ^1^H-NMR (CDC1_3_): *δ* 0.98 (s, 9H, C(CH_3_)_3_), 1.07 (t, 6H, *J* = 6.9 Hz, 2CH_3_), 3.75-4.06 (m, 2H, NCH_2_), 4.10-4.46 (m, 4H, 2OCH_2_), 4.93 (d, 1H, *J *= 8.6 Hz, CH(*t*-Bu)), 6.26 (s, 1H, PCH), 6.47 (s, 1H, NH), 7.15-7.47 (m, 10H, ArH), 7.92 (s, 1H, NH), 9.03 (br s, 1H, NH); ^13^C-NMR (CDCl_3_): *δ* 184.3, 170.7, 138.2, 135.4, 128.7, 128.0, 127.4, 66.3, 64.1, 55.4, 43.4, 34.8, 26.9; 16.5; ^31^P-NMR (CDCl_3_): *δ* 21.7; IR (KBr, cm^-1^): *v* 3292, 3068, 2965, 2867, 1669, 1562, 1221, 1013; Anal. Calcd for C_25_H_36_N_3_O_4_PS: C 59.39, H 7.18, N 8.31; Found: C 59.52, H 7.34, N 8.12. 

*Dipropyl (3-(L-1-benzylamino-3,3-dimethyl-1-oxobutan-2-yl)thioureido)(phenyl)methylphosphonate* (**3f**). White crystals, yield 93%; m.p. 171-172 ºC; [*α*]^20^*_D_*+9.6º (*c *0.1, acetone); ^1^H-NMR (CDC1_3_): *δ* 0.76 (t, 6H, *J *= 6.85 Hz, 2CH_3_), 1.03 (s, 9H, 3CH_3_), 1.42-1.74 (m, 4H, 2CH_2_), 3.70-3.95 (m, 2H, NCH_2_), 4.09-4.47 (m, 4H, 2OCH_2_), 4.94 (d, 1H, *J *= 8.6 Hz, CH(*t*-Bu)), 6.25 (s, 1H, PCH), 6.53 (s, 1H, NH), 7.12-7.47 (m, 10H, ArH), 7.94 (s, 1H, NH), 9.11 (br s, 1H, NH); ^13^C-NMR (CDC1_3_): *δ* 184.4, 170.6, 138.0, 135.2, 128.7, 128.4, 127.5, 69.4, 65.8, 55.3, 43.3, 34.7, 26.8, 24.0, 9.9; ^31^P-NMR (CDCl_3_): *δ* 21.5; IR (KBr, cm^-1^): *v* 3288, 3056, 2952, 2873, 1677, 1543, 1218, 1012; Anal. Calcd for C_27_H_40_N_3_O_4_PS: C 60.77, H 7.55, N 7.87; Found: C 60.52, H 7.71, N 7.59.

*Diisopropyl (3-(L-1-benzylamino-3,3-dimethyl-1-oxobutan-2-yl)thioureido)(phenyl)methylphos- phonate *(**3g**). White crystals, yield 96%; m.p. 176-177 ºC; [*α*]^20^*_D_*+7.2º (*c *0.1, acetone); ^1^H-NMR (CDC1_3,_ 500 MHz): *δ* 1.01 (s, 9H, 3CH_3_), 1.19 (d, 12H, *J *= 6.3 Hz, 4CH_3_), 4.22-4.46 (m, 2H, NCH_2_), 4.63-4.84 (m, 2H, 2OCH), 4.96 (d, 1H, *J *= 7.45 Hz, CH(*t*-Bu)), 6.32 (s, 1H, PCH), 6.38 (s, 1H, NH), 7.11-7.52 (m, 10H, ArH), 7.80 (s, 1H, NH), 9.05 (br s, 1H, NH); ^13^C-NMR (CDC1_3, _125 MHz): *δ* 184.6, 170.7, 138.3, 135.8, 128.9, 128.6, 127.4, 73.1, 66.4, 55.9, 43.2, 34.6, 27.0, 24.3; ^31^P-NMR(CDCl_3,_ 200 MHz): *δ* 19.7; IR (KBr, cm^-1^): *v* 3286, 3057, 2963, 2861, 1672, 1548, 1224, 1011; Anal. Calcd for C_27_H_40_N_3_O_4_PS: C 60.77, H 7.55, N 7.87; Found: C 60.91, H 7.34, N 7.48.

*Dibutyl (3-(L-1-benzylamino-3,3-dimethyl-1-oxobutan-2-yl)thioureido)(phenyl)methylphosphonate* (**3h**). White crystals, yield 91%; m.p. 122-124 ºC; [*α*]^20^*_D_*+12.1º (*c *0.1, acetone); ^1^H-NMR (CDC1_3_): *δ* 0.79 (t, 6H, *J *= 7.45 Hz, 2CH_3_), 0.96 (s, 9H, 3CH_3_), 1.16-1.71 (m, 8H, 2 CH_2_CH_2_), 3.71-4.01 (m, 2H, NCH_2_), 4.06-4.51 (m, 4H, 2OCH_2_), 4.92 (d, 1H, *J *= 8.6 Hz, CH(*t*-Bu)), 6.25 (s, 1H, PCH), 6.46 (s, 1H, NH), 7.13-7.46 (m, 10H, ArH), 7.92 (s, 1H, NH), 9.04 (br s, 1H, NH); ^13^C-NMR (CDC1_3_): *δ* 184.5, 170.6, 138.2, 135.6, 128.9, 128.6, 127.8, 67.7, 65.8, 55.5, 43.4, 34.8, 32.2, 26.9, 18.8, 13.7; ^31^P-NMR (CDCl_3_): *δ* 21.5; IR (KBr, cm^-1^): *v* 3283, 3065, 2954, 1671, 1552, 1216, 1013; Anal. Calcd for C_29_H_44_N_3_O_4_PS: C, 62.01; H, 7.90; N, 7.48; Found: C 62.24, H 7.63, N 7.69.

*Diethyl (3-(L-1-(2-fluorobenzylamino)-4-methyl-1-oxopentan-2-yl)thioureido)(phenyl)methyl-phosphonate* (**3i**). Colorless viscous liquid, yield 92%; [*α*]^20^*_D_*-22.3º (*c *0.1, acetone); ^1^H-NMR (CDCl_3_): *δ* 0.85 (d, 6H, *J* = 6.8 Hz, 2CH_3_), 1.11 (t, 6H, *J* = 5.7 Hz, 2CH_3_), 1.31-1.53(m, 2H, CH_2_), 1.67-1.74 (m, 1H, CH), 3.67-3.96 (m, 2H, NCH_2_), 4.26-4.47 (m, 4H, 2OCH_2_) , 5.20 (d, 1H, *J *= 6.7 Hz, CH), 6.29 (s, 1H, PCH), 6.32 (s, 1H, NH), 6.79-7.45 (m, 9H, ArH), 7.86 (s, 1H, NH), 8.57 (br s, 1H, NH); ^13^C-NMR (CDCl_3_): *δ* 183.5, 171.4, 159.8, 136.3, 131.3, 128.6, 127.2, 126.5, 124.1, 115.2, 63.9, 58.2, 54.4, 41.6, 34.1, 26.1, 23.3, 16.4; ^31^P-NMR (CDCl_3_):*δ* 22.7; ^19^F-NMR (CDCl_3_):*δ* -118.6; IR (KBr, cm^-1^): *v* 3290, 3085, 2977, 1662, 1557, 1216, 1025; Anal. Calcd for C_25_H_35_FN_3_O_4_PS: C 57.35, H 6.74, N 8.03; Found: C 57.53, H 6.55, N 8.28.

*Diisopropyl (3-(L-1-(2-fluorobenzylamino)-4-methyl-1-oxopentan-2-yl)thioureido)(phenyl)methyl-phosphonate* (**3j**). Colorless viscous liquid, yield 88%; [*α*]^20^*_D_*-17.2º (*c *0.1, acetone); ^1^H-NMR (CDCl_3_): *δ*0.87 (d, 18H, *J *= 6.4 Hz, 6CH_3_), 1.36-1.43 (m, 2H, CH_2_), 1.51-1.62 (m, H, CH), 3.75-4.16 (m, 2H, NCH_2_), 4.53-4.71 (m, 2H, 2OCH), 5.23 (d, 1H, *J *= 6.8 Hz, CH), 6.27 (s, 1H, PCH), 6.30 (s, 1H, NH), 6.82-7.43 (m, 9H, ArH), 7.74 (s, 1H, NH), 8.42 (br s, 1H, NH); ^13^C-NMR (CDCl_3_):*δ* 183.7, 171.2, 159.5, 136.4, 131.5, 128.8, 127.0, 126.7, 124.2, 115.3, 73.0, 59.1, 55.4, 41.4, 34.3, 26.6, 23.5, 16.3; ^31^P-NMR (CDCl_3_):*δ* 20.3; ^19^F-NMR (CDCl_3_):*δ* -118.8; IR (KBr, cm^-1^): *v* 3275, 3067, 2961, 1668, 1554, 1227, 1015; Anal. Calcd for C_27_H_39_FN_3_O_4_PS: C 58.78, H 7.13, N 7.62; Found: C 58.53, H 7.32, N 7.34.

*Diethyl (3-(L-1-(4-fluorobenzylamino)-4-methyl-1-oxopentan-2-yl)thioureido)(phenyl)methylphos-phonate* (**3k**). White solid, yield 94%; m.p. 31-32 ºC; [*α*]^20^*_D_*-16.7º (*c *0.1, acetone); ^1^H-NMR (CDCl_3_): *δ* 0.82 (d, 6H, *J* = 6.5 Hz, 2CH_3_), 1.09 (t, 6H, *J* = 5.8Hz, 2CH_3_), 1.27-1.52(m, 2H, CH_2_), 1.66-1.71 (m, 1H, CH), 3.71-3.99 (m, 2H, NCH_2_), 4.14-4.43 (m, 4H, 2OCH_2_) , 5.20 (d, 1H, *J *= 6.7 Hz, CH), 6.29 (s, 1H, PCH), 6.37 (s, 1H, NH), 6.74-7.45 (m, 9H, ArH), 8.01 (s, 1H, NH), 8.74 (br s, 1H, NH); ^13^C-NMR (CDCl_3_): *δ* 183.6, 171.7, 160.9, 137.1, 135.8, 128.7, 127.1, 126.6, 115.4, 64.1, 58.5, 54.2, 44.2, 41.3, 26.3, 23.6, 16.5; ^31^P-NMR (CDCl_3_):*δ* 22.2; ^19^F-NMR (CDCl_3_):*δ* -115.1 ppm; IR (KBr, cm^-1^): *v* 3285, 3080, 2963, 1676, 1551, 1223, 1020 cm^-1^; Anal. Calcd for C_25_H_35_FN_3_O_4_PS: C 57.35, H 6.74, N 8.03; Found: C 57.59, H 6.48, N 7.96

*Diisopropyl (3-(L-1-(4-fluorobenzylamino)-4-methyl-1-oxopentan-2-yl)thioureido)(phenyl)methyl-phosphonate* (**3l**). White solid, yield 90%; m.p. 43-44 ºC; [*α*]^20^*_D_*-13.3º (*c *0.1, acetone); ^1^H-NMR (CDCl_3_): *δ* 0.84 (d, 18H, *J *= 6.3 Hz, 6CH_3_), 1.39-1.57 (m, 2H, CH_2_), 1.64-1.69 (m, H, CH), 4.21-4.43 (m, 2H, NCH_2_), 4.63-4.72 (m, 2H, 2OCH), 5.19 (d, 1H, *J *= 5.8 Hz, CH), 6.25 (s, 1H, PCH), 6.35 (s, 1H, NH), 6.95-7.40 (m, 9H, ArH), 7.98 (s, 1H, NH), 8.71 (br s, 1H, NH); ^13^C-NMR (CDC1_3_): *δ* 183.7, 172.1, 161.2, 137.3, 135.6, 128.8, 127.4, 126.8, 115.3, 73.3, 59.6, 55.7, 43.4, 40.8, 26.4, 24.5, 23.2; ^31^P-NMR (CDCl_3_): *δ* 21.9; ^19^F-NMR (CDCl_3_):*δ* -115.4; IR (KBr, cm^-1^): *v* 3287, 3072, 2968, 1678, 1548, 1220, 1008; Anal. Calcd for C_27_H_39_FN_3_O_4_PS: C 58.78, H 7.13, N 7.62; Found: C 58.64, H 7.35, N 7.41.

*Diethyl (3-(L-4-methyl-1-oxo-1-(4-(trifluoromethyl)phenylamino)pentan-2-yl)thioureido)(phenyl)-methylphosphonate* (**3m**). Colorless viscous liquid, yield 84%; [*α*]^20^*_D_*-8.9º (*c *0.1, acetone); ^1^H-NMR (CDCl_3_): *δ*0.94 (d, 6H, *J* = 5.7 Hz, 2CH_3_), 1.13 (t, 6H, *J *= 6.9 Hz, 2CH_3_), 0.99-1.02(m, H, CH), 1.66-1.72 (m, 2H, CH_2_), 3.92-4.13 (m, 4H, 2OCH_2_), 5.03-5.11 (m, 1H, CH), 6.31 (s, 1H, PCH), 7.31-7.79 (m, 9H, ArH), 10.07 (s, 1H, NH); ^13^C-NMR (CDCl_3_): *δ* 183.8, 174.2, 141.7, 136.9, 128.4, 127.0, 126.5, 126.1, 125.7, 123.3, 119.8, 73.4, 58.2, 54.1, 41.4, 26.2, 23.4, 21.8; ^31^P NMR (CDCl_3_): *δ* 22.3; ^19^F-NMR (CDCl_3_): *δ* -63.4; IR (KBr, cm^-1^): *v* 3467, 3280, 2968, 1648, 1552, 1218, 1026; Anal. Calcd for C_25_H_33_F_3_N_3_O_4_PS: C 53.66, H 5.94, N 7.51; Found: C 53.74, H 6.17, N 7.22.

*Diisopropyl(3-(L-4-methyl-1-oxo-1-(4-(trifluoromethyl)phenylamino)pentan-2-yl)thioureido) (phenyl -methylphosphonate* (**3n**). Colorless viscous liquid, yield 79%; [*α*]^20^*_D_***-**10.5º (*c *0.1, acetone); ^1^H-NMR (CDCl_3_): *δ* 0.98 (d, 18H, *J *= 6.4 Hz, 6CH_3_), 1.36-1.42 (m, 1H, CH), 1.63-1.68 (m, H, CH_2_), 4.17-4.43 (m, 2H, 2OCH), 5.06-5.16 (m, 1H, CH), 6.33 (s, 1H, PCH), 7.29-7.74 (m, 9H, ArH), 10.12 (br s, 1H, NH); ^13^C-NMR (CDC1_3_): *δ* 183.9, 174.4, 141.5, 137.2, 128.5, 127.1, 126.8, 126.2, 125.8, 123.5, 119.4, 73.6, 58.5, 54.2, 41.3, 26.1, 23.2, 21.7; ^31^P-NMR (CDCl_3_): *δ* 22.5; ^19^F-NMR (CDCl_3_): *δ* -63.2; IR (KBr, cm^-1^): *v* 3465, 3276, 2962, 1645, 1546, 1212, 1028; Anal. Calcd for C_27_H_37_F_3_N_3_O_4_PS: C 55.19, H 6.35, N 7.15; Found: C 55.26, H 6.71, N 6.97.

### 3.5. Antiviral Biological Assays

#### 3.5.1. Purification of TMV

Using Gooding’s method [16], the upper leaves of *Nicotiana tabacum *L inoculated with TMV were selected and were grounded in phosphate buffer, then filtered through double layer pledget. The filtrate was centrifuged at 10,000 g, treated twice with PEG, and centrifuged again. The whole experiment was processed at 4 ºC. Absorbance value was estimated at 260 nm by ultraviolet spectrophotometer:

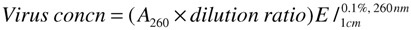


#### 3.5.2. Protection effect of compound against TMV *in vivo*

The compound solution was smeared on the left side while solvent served as the control on the right side of *Nicotiana tabacum. *L leaves of the same ages. The leaves were then inoculated by the virus after 12 h. Tobacco mosaic virus of 6 × 10^-3 ^mg/mL was dipped with brush to inoculate on the leaves which were previously scattered with silicon carbide. Then the leaves were washed with water and rubbed softly along the nervure once or twice. The local lesion numbers appeared 3-4 days after inoculation [17]. Triplicate repetitions were conducted for each compound.

#### 3.5.3. Curative effect of compound against TMV *in vivo*

The leaves on *Nicotiana tabacum. *L of the same ages were selected. The tobacco mosaic virus with 6 ´10^-3 ^mg/mL concentration was dipped and inoculated on the whole leaves. Then the leaves were washed with water and dried. The compound solution was smeared on the left side and the solvent was smeared on the right side for control. The local lesion numbers were then recorded 3-4 days after inoculation [17]. For each compound, three repetitions were conducted to ensure the reliability of the results:

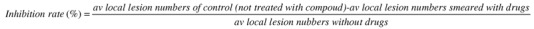


## 4. Conclusions

A series of novel chiral thioureas containing *α*-aminophosphonate moieties were synthesized in high yield under mild conditions by the addition in THF of *O*, *O*′-dialkyl isothiocyanato(phenyl)-methylphosphonates **8** to L-leucine derivatives. The bioassay results showed that title compounds exhibited moderate to good anti-TMV bioactivity. In particular, compounds **3l** (R_1_= *p*-FC_6_H_4_CH_2_, R_2_= *i*-Bu, R= *i*-Pr) and **3n** (R_1_= *p*-CF_3_C_6_H_4_, R_2_= *i*-Bu, R= *i*-Pr) showed better biological activity than other structurally related analogues. The L-leucyl and phosphonate moiety derived from branched alkyl chains are preferred over straight chain alkylated ones in the design of the title thioureas, moreover, the compounds bearing an electron withdrawing group (R_1_= *p*-F, *p*-CF_3_) at the 4-position of the aromatic ring showed better anti-TMV activities. At for the absolute configuration, compound **L-3l** (derived from L-leucine) showed more significant activity against TMV than its enantiomer **D-3l** and the racemate (**±)-3l**. The present work demonstrates that the antiviral activity of chiral thioureas was significantly improved through introduction of suitably substituted derivatives with L-configuration. The influence of different substituents, minor structural modifications and steric parameters on structure activity relationships for identifying lead bioactive compounds will be studied in our future investigations.
